# Herbicide drift exposure leads to reduced herbicide sensitivity in *Amaranthus* spp.

**DOI:** 10.1038/s41598-020-59126-9

**Published:** 2020-02-07

**Authors:** Bruno C. Vieira, Joe D. Luck, Keenan L. Amundsen, Rodrigo Werle, Todd A. Gaines, Greg R. Kruger

**Affiliations:** 1 West Central Research and Extension Center, University of Nebraska-Lincoln, North Platte, NE USA; 2 Department of Biological Systems Engineering, University of Nebraska-Lincoln, Lincoln, NE USA; 3 Department of Agronomy and Horticulture, University of Nebraska-Lincoln, Lincoln, NE USA; 4 Department of Agronomy, University of Wisconsin–Madison, Madison, WI USA; 5 Department of Bioagricultural Sciences and Pest Management, Colorado State University, Fort Collins, CO USA

**Keywords:** Environmental sciences, Plant sciences, Plant stress responses

## Abstract

While the introduction of herbicide tolerant crops provided growers new options to manage weeds, the widespread adoption of these herbicides increased the risk for herbicide spray drift to surrounding vegetation. The impact of herbicide drift in sensitive crops is extensively investigated, whereas scarce information is available on the consequences of herbicide drift in non-target plants. Weeds are often abundant in field margins and ditches surrounding agricultural landscapes. Repeated herbicide drift exposure to weeds could be detrimental to long-term management as numerous weeds evolved herbicide resistance following recurrent-selection with low herbicide rates. The objective of this study was to evaluate if glyphosate, 2,4-D, and dicamba spray drift could select *Amaranthus* spp. biotypes with reduced herbicide sensitivity. Palmer amaranth and waterhemp populations were recurrently exposed to herbicide drift in a wind tunnel study over two generations. Seeds from survival plants were used for the subsequent rounds of herbicide drift exposure. Progenies were subjected to herbicide dose-response studies following drift selection. Herbicide drift exposure rapidly selected for *Amaranthus* spp. biotypes with reduced herbicide sensitivity over two generations. Weed management programs should consider strategies to mitigate near-field spray drift and suppress the establishment of resistance-prone weeds on field borders and ditches in agricultural landscapes.

## Introduction

Current weed management practices such as herbicide applications tend to focus on property-level management decisions, where actions usually neglect landscape-scale outcomes^1^. The recurrent herbicide drift complaints in agricultural landscapes evidences this situation. After the introduction of glyphosate tolerant crops in the late nineties, and the recent introduction of 2,4-D and dicamba tolerant crops, growers gained new herbicide options and flexibility to manage weeds^2–4^. However, the widespread adoption of these herbicides in weed management programs increased the risk of off-target movement associated with herbicide applications. Spray drift is the part of the pesticide application deflected away from the target area during or following applications^5^. Glyphosate, 2,4-D, and dicamba spray drift have been reported to cause severe injury on sensitive vegetation and crops, especially when best practices are not adopted during applications^6–13^.

While the consequences of herbicide drift towards sensitive crops are extensively investigated in the literature, scarce information is available on the consequences of herbicide drift towards other plant communities surrounding agricultural landscapes^14^. Weed species such as waterhemp [*Amaranthus tuberculatus* (Moq.) J. D. Sauer] and Palmer amaranth (*Amaranthus palmeri* S. Wats.) are often abundant in field margins and ditches throughout the US^15–17^. Herbicide drift exposure could be detrimental to long-term weed management as numerous weed species have evolved herbicide resistance following recurrent applications of low herbicide rates^18–28^, and we reported in a previous study that spray drift can expose weeds to herbicide doses associated with herbicide resistance selection^14^. In addition, herbicide resistance has been widely reported in weed populations inhabiting field margins and ditches surrounding agricultural landscapes^15,17,29^.

Recurrent selection to low herbicide doses can gradually select for metabolism alleles present within the standing genetic variation of the population, which can progressively lead to herbicide resistance on weeds^20,30–32^. Some researchers also suggest that low rates of herbicides could induce new stress-related mutations and epigenetic alterations on weeds, ultimately leading to reduced herbicide sensitivity^33–35^.

Recombination and accumulation of minor resistance alleles can occur at a faster rate in cross-pollinated species, such as Palmer amaranth and waterhemp, during recurrent selection with low rates of herbicides^21,22,36^. These C4 summer annual *Amaranthus* spp. are among the most troublesome weed species in the US row crop production systems^37^. Both are obligate outcrossing dioecious weed species with a fast growth habitat, extended emergence window, and prolific seed production with high genetic plasticity which pose a challenge to their management^37–44^. Numerous Palmer amaranth and waterhemp populations have evolved resistance to herbicides that target 5-enolpyruvylshikimate-3-phosphate synthase (EPSPS), 4-hydroxyphenylpyruvate dioxygenase (HPPD), photosystem II, protoporphyrinogen oxidase (PPO), auxin receptors, microtubule assembly, and acetolacte synthase (ALS) in the US^15,17,45–54^. Moreover, pollen mediated gene flow has been reported as a major contributor to herbicide resistance dissemination in Palmer amaranth and waterhemp in the US Midwest^55,56^.

Although controlling weed populations on field margins and ditches is considered a best management practice to delay herbicide resistance evolution, these weed populations are often neglected in agricultural landscapes^15–17,29^. The hypothesis of this study is that repeated herbicide drift in field borders and ditches can select weed biotypes with reduced herbicide sensitivity. Therefore, the objective of this study was to evaluate whether drift from glyphosate, 2,4-D, and dicamba applications could select for *Amaranthus* spp. with reduced herbicide sensitivity over two generations in a wind tunnel study.

## Material and Methods

### Plant material

Palmer amaranth and waterhemp seeds were collected from 10–20 putative herbicide susceptible plants in wheat (*Triticum aestivum* L.) and corn (*Zea mays* L.) fields in Nebraska (Table 1). Seeds from within a single field were identified as a population (Chase and Perkins for Palmer amaranth, and Thayer and Stanton for waterhemp) and stored at −20 °C for a minimum of three months to overcome dormancy. Following sowing, seedlings were transplanted into plastic tubes (1 L) containing commercial potting mix (Berger BM7 Bark Mix, Saint Modeste, QC, Canada) and maintained under greenhouse conditions (30/20 °C [day/night] with a 16 h photoperiod) at the Pesticide Application Technology Laboratory (University of Nebraska-Lincoln, West Central Research and Extension Center, North Platte, NE)^17^. Supplemental light (LED growth lights 520 μmol s^−1^, Philips Lighting, Somerset, NJ, USA) was provided to ensure a 16-h photoperiod. Plants were supplied with water including fertilizer solution (0.2% v/v) as needed (UNL 5-1-4, Wilbur-Ellis Agribusiness, Aurora, CO, USA)^17^.

**Table 1:** Palmer amaranth and waterhemp populations from Nebraska used in the herbicide spray drift selection study.

Species	County	Crop	Year
Palmer amaranth	Chase	corn	2014
Palmer amaranth	Perkins	wheat	2015
Waterhemp	Thayer	corn	2014
Waterhemp	Stanton	corn	2014

### Herbicide drift recurrent selection

Herbicide drift simulations were conducted in the low speed wind tunnel at the Pesticide Application Technology Laboratory. Glyphosate, 2,4-D, and dicamba solutions were prepared at 140 L ha^−1^ carrier volume (Table 2). The glyphosate solution had the addition of ammonium sulfate at 5% v/v to overcome antagonistic effects of cationic salts in hard water (Bronc, Wilbur-Ellis Agribusiness, Aurora, CO, USA). Herbicide applications were performed at 140 L ha^−1^ with two even nozzles, a conventional flat-fan nozzle (TP95015EVS) and an air-inclusion (AI) nozzle (AI95015EVS) (TeeJet Technologies Spraying Systems Co., Glendale Heights, IL, USA) at 230 kPa with constant wind speed of 4.47 m s^−1^ as we described in a previous study^14^. Nozzles were selected to provide high (Fine spray classification) and low (Ultra Coarse spray classification) drift potentials. The average air temperature and relative humidity during applications were 25 °C and 45%, respectively. Palmer amaranth and waterhemp plants (15–20 cm-tall) were positioned at four downwind distances: 1.0, 1.5, 2.0, 2.5 m from the nozzle simulating plants inhabiting field margins. Eighty plants of each population were exposed to herbicide*nozzle drift treatments, with 20 plants per distance. During applications, nozzles were positioned at 70 cm from the ground. Following herbicide drift exposure, plants were returned and kept under greenhouse conditions as previously described. Plant mortality was evaluated at 35 days after treatment (DAT).

**Table 2:** Herbicide solutions, rates (grams of acid equivalent per hectare), and product manufacturers for solutions used in the herbicide spray drift study^a^.

Herbicide	Active ingredient	Product manufacturer	Rate
Clarity	Dicamba diglycolamine salt	BASF Corporation, Research, Triangle Park, NC, USA	560 g ae ha^−1^
Roundup PowerMax	Glyphosate potassium salt	Bayer CropScience, Research, Triangle Park, NC, USA	867 g ae ha^−1^
Weedar 64	2,4-D dimethylamine salt	Nufarm Inc, Alsip, IL, USA	1064 g ae ha^−1^

^a^Glyphosate solution had the addition of ammonium sulfate solution at 5% v/v (Bronc, Wilbur-Ellis Agribusiness, Aurora, CO, USA).

Survivors of each herbicide*nozzle*population treatment were enclosed within tents (plants from all distances were pooled) constructed with 213-cm by 152-cm pollination bags (Vilutis & Co., Frankfort, IL, USA) to ensure cross-pollination exclusively within specific treatments. Tents were periodically shaken to facilitate pollination. Seeds from all plants within each treatment were collected at maturity, pooled, and termed P_1_ seeds. Seeds were dried at greenhouse room temperature and stored at −20 °C for 15 days. P_1_ seeds of each herbicide*nozzle*population treatment were used for the subsequent round of herbicide drift selection. Plant material, herbicide drift treatments, and isolation on pollination tents were conducted as previously described, and survivors from the second herbicide drift selection were grown to seed to establish the P_2_ progeny for each treatment. During each herbicide drift selection (P_1_ and P_2_ selection), a group of 40 untreated plants per population (Chase, Perkins, Thayer, and Stanton) was maintained and isolated on pollination tents using the same procedure previously described to establish P_1_ and P_2_ unselected controls. The study had a factorial arrangement with weed species, nozzle, and herbicide as factors in a completely randomized design. Plant mortality of treatment combinations were analyzed with a Beta-binomial distribution using a logit link function with a generalized mixed model in SAS software (SAS v9.4, SAS Institute Inc., Cary, NC, USA) and comparisons among treatments were performed using Fisher’s least significant difference procedure at significance level α = 0.05.

### Herbicide dose response

Palmer amaranth and waterhemp P_2_ progenies (herbicide*population*nozzle treatments and non-selected controls) were subjected to glyphosate, 2,4-D, and dicamba dose-response study (respective to herbicide drift selection treatment) in the Pesticide Application Technology Laboratory. Following sowing, seedlings from P_2_ progenies were transplanted into plastic tubes containing commercial potting mix and maintained under greenhouse conditions as previously described. Plants (10- to 12-cm tall) were sprayed with different glyphosate, 2,4-D, and dicamba rates (Table 3) using a research spray chamber (DeVries, Hollandale, MN, USA) calibrated to deliver 93.5 L ha^−1^ using an AI95015EVS nozzle at 414 kPa. The experiment was conducted in a randomized complete design with four replications per treatment in which a single plant was considered as an experimental unit. Plant above ground biomass was harvested at 30 DAT and oven dried at 65 °C to constant weight. Biomass data were converted into percentage of biomass reduction as compared to the untreated control. A non-linear regression model was fitted to dry weight data in response to herbicide dose using the *drc* package in R software (R Foundation for Statistical Computing, Wien, Austria)^17,57^. The effective-dose to reduce 90% of plant biomass (GR_90_) was estimated for each P_2_ progeny using a four-parameter log-logistic model: *y* = *c* + *{d − c/1* + *exp[b(log x − log e)]}* in which y corresponds to the biomass reduction (%), *b* is the slope at the inflection point, *c* is the lower limit of the model (fixed to 0%), *d* is the upper limit (fixed to 100%), and *e* is the inflection point (effective dose to reduce plant biomass in 50%). Resistance ratios were calculated as the ratio of the GR_90_ for each selected P_2_ population to the respective P_2_ unselected population^17^. The experiment was conducted twice and data were combined.

**Table 3:** Herbicide rates (grams of acid equivalent per hectare) used in the dose response study with P_2_ Palmer amaranth and waterhemp plants^a^.

Herbicide	Doses (g ae ha^−1^)	
	Palmer amaranth	waterhemp
glyphosate	3.9, 9.9, 19.7, 39.4, 197, 394.0, 985.1, and 1970.2	3.9, 9.9, 19.7, 39.4, 394.0, 985.1, and 1970.2
2,4-D	166.4, 332.8, 831.9, and 1663.8	33.3, 83.2, 166.4, 332.8, 831.9, and 1663.8
dicamba	3.5, 8.8, 17.5, 350.3, 875.7, and 1751.3	35, 87.6, 175.1, 350.3, 875.7, and 1751.3

^a^Glyphosate solution had the addition of ammonium sulfate solution at 5% v/v (Bronc, Wilbur-Ellis Agribusiness, Aurora, CO, USA).

## Results and Discussion

### Herbicide drift exposure

Glyphosate, 2,4-D, and dicamba drift exposure resulted in Palmer amaranth and waterhemp mortality (Tables 4 and 5). *Amaranthus* spp. mortality was influenced by nozzle type (*p* < 0.0001) and herbicide by weed species interaction (*p* < 0.0001). Herbicide drift from the flat fan nozzle resulted in 54–69% (CI 95%) overall mortality when the other variables were pooled, whereas the air inclusion nozzle resulted in 19 to 32% (CI 95%). These results corroborate previous field and wind tunnel results where applications with air inclusion nozzles resulted in less particle drift compared to flat fan nozzles^58–62^. The preorifice component of air inclusion nozzles is designed to reduce the solution pressure as it exits the nozzle, thereby increasing the droplet size of the spray and consequently reducing the drift potential^63,64^. We reported in a previous study that herbicide applications in a wind tunnel (4.47 m s^−1^ wind speed) with the flat fan nozzle resulted in 32, 23, 17, and 14% of herbicide drift (in relation to volume sprayed) at 1, 1.5, 2.0, and 2.5 m from the nozzle, respectively, whereas applications with the air inclusion nozzle resulted in 11, 7, 5, and 3% herbicide drift in the same downwind distances^14^.

**Table 4:** Combined mortality of Palmer amaranth progenies following herbicide drift exposure in a wind tunnel study^a^.

Population	Progeny	Nozzle	Herbicide drift		
			glyphosate	2,4-D	dicamba
			Mortality (%)		
Perkins	P_0_	air inclusion	67.5	11.3	18.8
		flat fan	93.75	42.5	62.5
	P_1_	air inclusion	23.5	5	10.5
		flat fan	†	26.75	44.5
Chase	P_0_	air inclusion	76.25	13.75	23.25
		flat fan	96.25	61.25	61.25
	P_1_	air inclusion	100	12.5	2.75
		flat fan	†	21.25	55

^a^Total of 80 plants per population*progeny*nozzle*herbicide*, with 20 plants per distance (1.0, 1.5, 2.0, and 2.5 m from the nozzle).

^†^Progenies were not established.

**Table 5:** Combined mortality of waterhemp progenies following herbicide drift exposure in a wind tunnel study^a^.

Population	Progeny	Nozzle	Herbicide drift		
			glyphosate	2,4-D	dicamba
			Mortality (%)		
Thayer	P_0_	air inclusion	31.3	21.3	15
		flat fan	50	57.5	50
	P_1_	air inclusion	28.8	33.8	2.5
		flat fan	27.5	82.5	48.75
Stanton	P_0_	air inclusion	8.75	7.5	3.75
		flat fan	30	80	26.25
	P_1_	air inclusion	15	25	8.75
		flat fan	39	86.25	55

^a^Total of 80 plants per population*progeny*nozzle*herbicide*, with 20 plants per distance (1.0, 1.5, 2.0, and 2.5 m from the nozzle).

Glyphosate drift resulted in increased plant mortality on Palmer amaranth (81–95% CI 95%) compared to 2,4-D (16–36% CI 95%) and dicamba (23–45% CI 95%). 2,4-D drift resulted in higher plant mortality on waterhemp (37–61% CI 95%) compared to glyphosate (18–41% CI 95%) and dicamba (16–36% CI 95%). Both species had similar mortality when exposed to dicamba drift. Inherent differences in herbicide response between Palmer amaranth and waterhemp were reported in previous studies, with Palmer amaranth being more tolerant to PPO-inhibitors^50^, but more susceptible to glyphosate^17^. Palmer amaranth was very susceptible to glyphosate drift, especially with applications using the flat fan nozzle. As a result, P_1_ and P_2_ progenies were not established for both Palmer amaranth populations (Perkins and Chase) exposed to glyphosate drift with the flat fan nozzle. Although a P_1_ progeny was established for the Chase population exposed to glyphosate drift using the air inclusion nozzle, a P_2_ progeny was not established as plants did not survive the second round of herbicide drift exposure.

A previously susceptible Palmer amaranth population evolved levels of glyphosate resistance following four selection rounds with low rates of glyphosate, with 58, 43, 51, and 79% plant mortality during selection rounds^26^. Similar resistance shift results were reported in an annual ryegrass (*Lolium rigidum* Gaudin) population recurrently selected with low rates of glyphosate in field conditions, although higher plant mortality ranging from 71 to 90% during four selection rounds was observed^22^. A wild radish (*Raphanus raphanistrum* L.) population evolved levels of 2,4-D resistance after four rounds of selection with sublethal rates of 2,4-D, with 71, 88, 77, and 76% mortality during each selection round^18^. Similarly, a Palmer amaranth population evolved higher levels of dicamba resistance after recurrent selection with low dicamba rates, with 47, 68, 29, and 79% plant mortality during each of the selection rounds^27^.

### Herbicide drift recurrent selection

**Figure 1:**
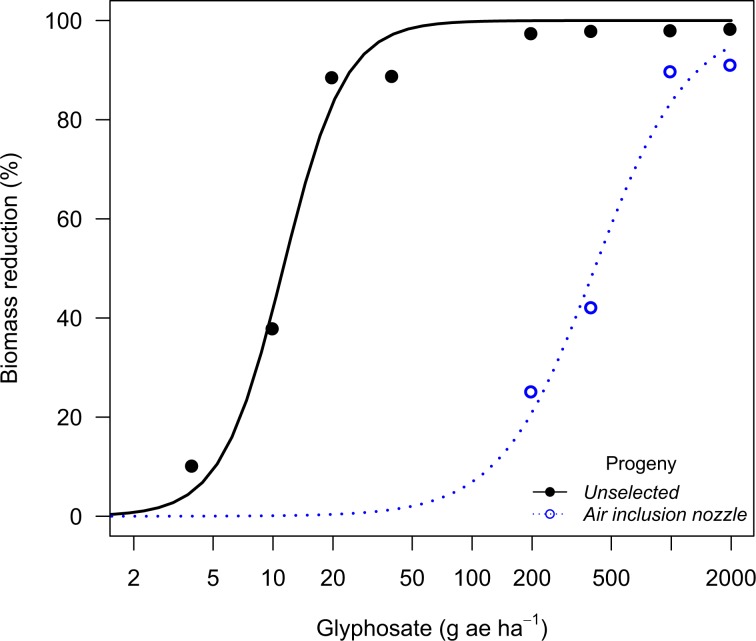
Biomass reduction for the Palmer amaranth population (P_2_) from Perkins County (NE) following recurrent selection to glyphosate spray drift at 30 days after treatment in the glyphosate dose response study.

**Figure 2:**
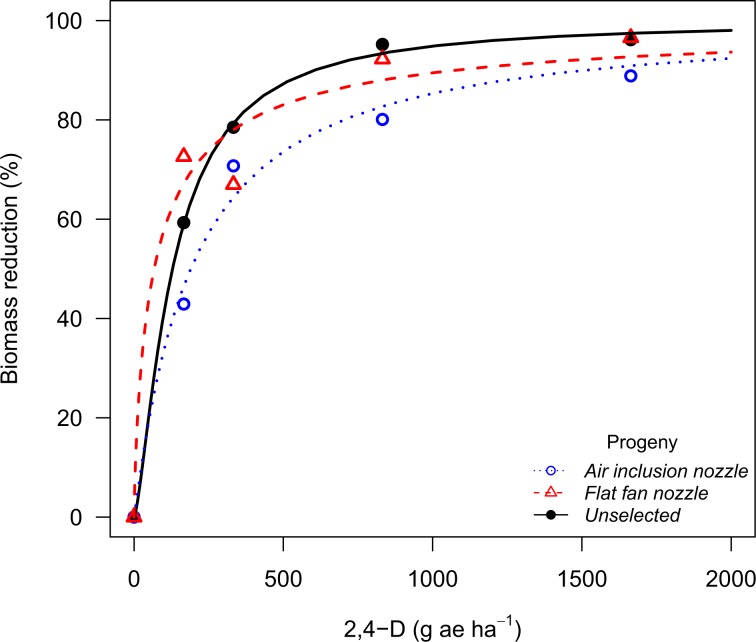
Biomass reduction for the Palmer amaranth population (P_2_) from Perkins County (NE) following recurrent selection to 2,4-D spray drift at 30 days after treatment in the 2,4-D dose response study.

**Figure 3:**
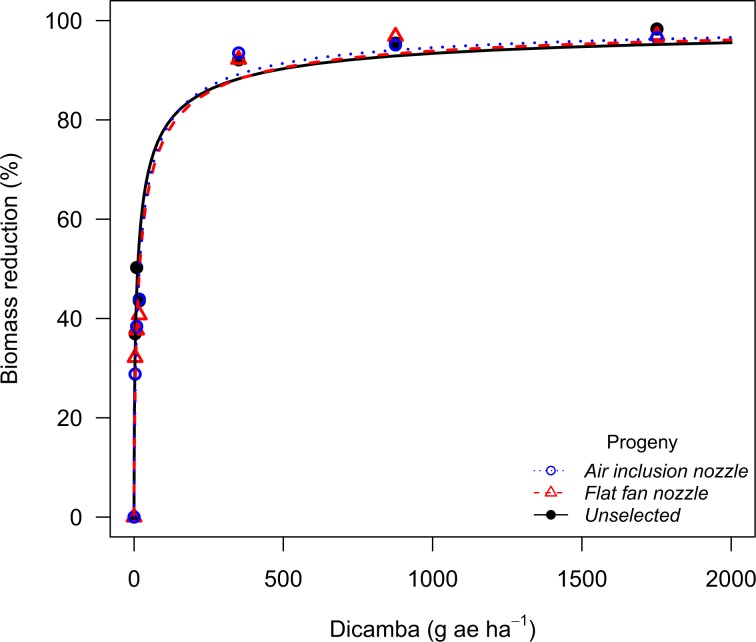
Biomass reduction for the Palmer amaranth population (P_2_) from Chase County (NE) following recurrent selection to dicamba spray drift at 30 days after treatment in the dicamba dose response study.

The Palmer amaranth population from Perkins County evolved glyphosate resistance (54.7-fold in the GR_90_) after being recurrently exposed to glyphosate drift with the air inclusion nozzle (Fig. 1). The Perkins population exposed to 2,4-D drift with the air inclusion nozzle had 2.5-fold shift in the GR_90_ after two selection rounds, whereas the progeny exposed to 2,4-D drift with the flat fan nozzle had a 1.8-fold shift (Fig. 2). On the other hand, the Palmer amaranth population from Chase County had no resistance shift after being recurrently selected with 2,4-D drift with both air inclusion and flat fan nozzles (Table 6). Moreover, both Palmer amaranth populations had no sensitivity shift following dicamba drift selection with both air inclusion and flat fan and nozzles (Fig. 3).

**Table 6:** Log-logistic model parameter estimates, standard errors, dose to 90% biomass reduction (GR_90_), and resistance ratio (R/S) for each P_2_ population of Palmer amaranth^a^.

Population	Herbicide	Progeny	*b*	*e*	GR_90_	R/S
Perkins	Glyphosate	Unselected	−1.7 ± 0.4	11.2 ± 0.4	24.6 ± 2.3	—
		Air inclusion	−2.8 ± 0.3	376.0 ± 45.4	1346.0 ± 376.5	54.7
	2,4-D	Unselected	−1.4 ± 0.3	128.8 ± 20.0	603.8 ± 143.4	—
		Air inclusion	−1.1 ± 0.2	190.0 ± 24.2	1506.6 ± 440.1	2.5
		Flat Fan	−0.8 ± 0.2	67.3 ± 25.5	1073.3 ± 372.1	1.8
	Dicamba	Unselected	−0.7 ± 0.1	25.0 ± 2.9	558.9 ± 154.2	—
		Air inclusion	−0.7 ± 0.1	19.4 ± 2.1	393.9 ± 117.0	0.7
		Flat Fan	−0.6 ± 0.1	12.4 ± 1.5	427.2 ± 126.7	0.8
Chase	2,4-D	Unselected	−1.2 ± 0.2	131.5 ± 16.4	781.0 ± 150.1	—
		Air inclusion	−1.3 ± 0.2	126.8 ± 16.9	657.2 ± 140.0	0.8
		Flat Fan	−1.1 ± 0.2	135.9 ± 17.2	932.1 ± 189.8	1.2
	Dicamba	Unselected	−0.6 ± 0.1	12.1 ± 1.4	470.4 ± 139.8	—
		Air inclusion	−0.7 ± 0.1	17.8 ± 2.0	394.8 ± 112.7	0.8
		Flat Fan	−0.7 ± 0.1	18.4 ± 2.0	457.6 ± 124.4	1.0

^a^*b* parameter corresponds to the slope at the inflection point; *e* parameter corresponds to the inflection point; GR_90_ corresponds to the effective dose to reduce plant biomass by 90%; resistance ratios (R/S) were calculated as the ratio of the GR_90_ for each P_2_ population to the respective P_2_ unselected population.

**Figure 4:**
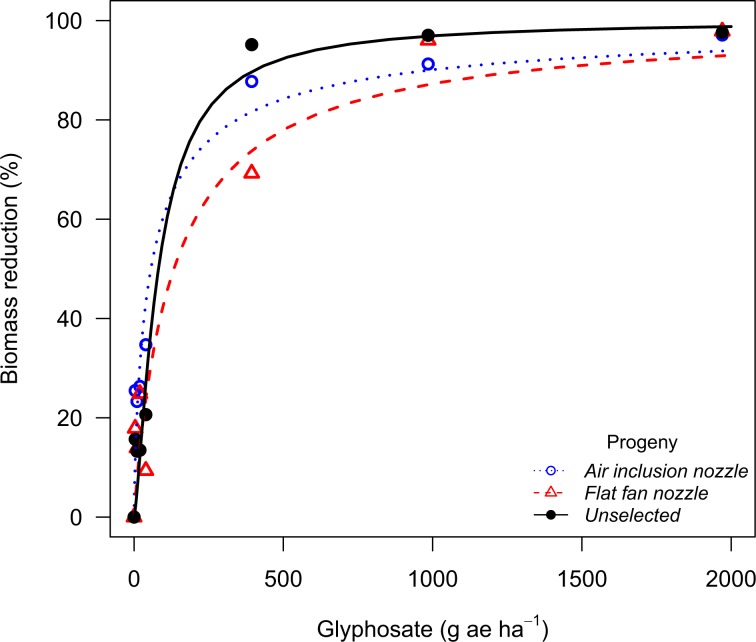
Biomass reduction for the waterhemp population (P_2_) from Thayer County (NE) following recurrent selection to glyphosate spray drift at 30 days after treatment in the glyphosate dose response study.

**Figure 5:**
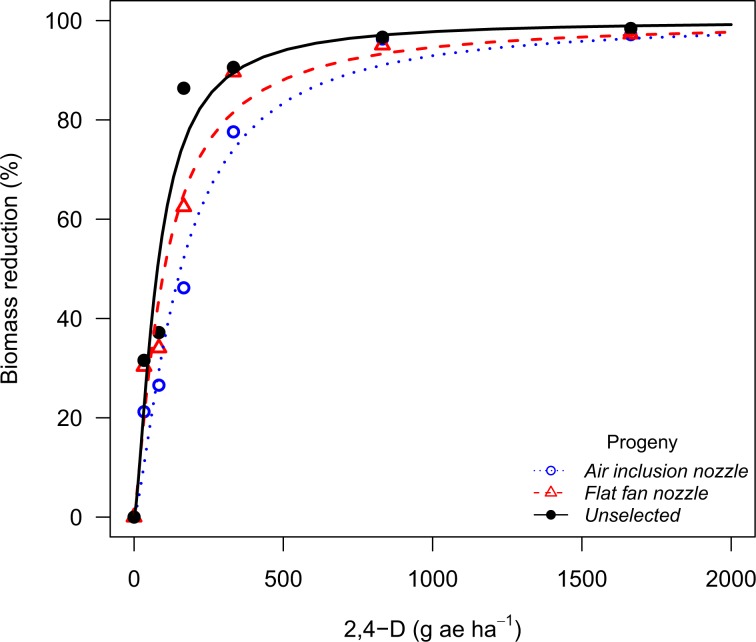
Biomass reduction for the waterhemp population (P_2_) from Thayer County (NE) following recurrent selection to 2,4-D spray drift at 30 days after treatment in the 2,4-D dose response study.

**Figure 6:**
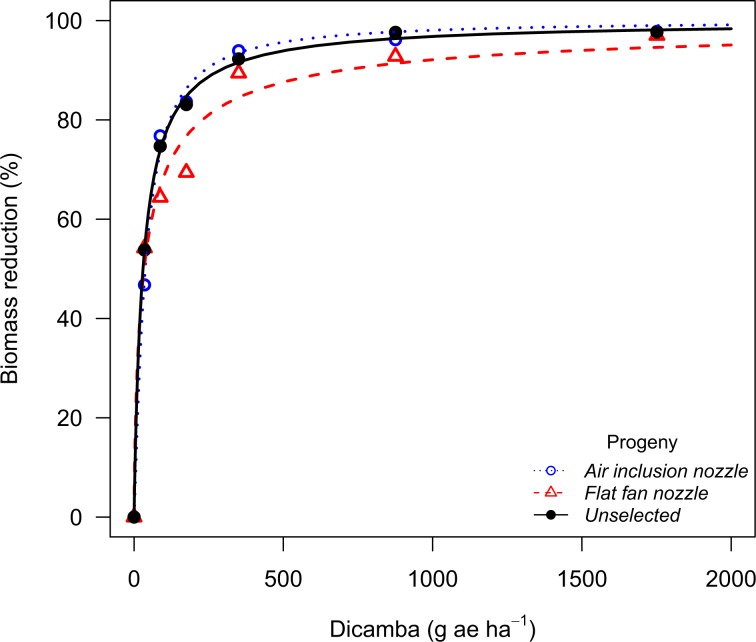
Biomass reduction for the waterhemp population (P_2_) from Stanton County (NE) following recurrent selection to dicamba spray drift at 30 days after treatment in the dicamba dose response study.

The waterhemp population from Stanton County showed no evidence of resistance shift when recurrently selected with glyphosate drift with the air inclusion nozzle, whereas plants exposed to glyphosate drift with the flat fan nozzle had a 2-fold glyphosate resistance shift (Table 7). The Thayer population had a 2.4 and 3.3-fold glyphosate resistance shift after being recurrently exposed to glyphosate drift with the air inclusion and the flat fan nozzles, respectively (Fig. 4). The Thayer population also had its 2,4-D sensitivity reduced after recurrent exposure to 2,4-D drift using the air inclusion (2.2-fold) and the flat fan nozzle (1.7-fold), whereas no shifts were observed for the Stanton population (Fig. 5). Recurrent exposure to dicamba drift with the air inclusion and the flat fan nozzles resulted in dicamba sensitivity shifts in the Thayer population (1.5 and 2.2-fold shift, respectively). The Stanton population also had its sensitivity to dicamba increased, but only for progenies exposed to dicamba drift with the flat fan nozzle (2.4-fold) (Fig. 6).

**Table 7:** Log-logistic model parameter estimates, standard errors, dose to 90% biomass reduction (GR_90_), and resistance ratios (R/S) for each P_2_ population of waterhemp^a^.

Population	Herbicide	Progeny	*b*	*e*	GR_90_	R/S
Stanton	Glyphosate	Unselected	−1.8 ± 0.3	101.4 ± 17.8	349.0 ± 109.2	—
		Air inclusion	−1.1 ± 0.1	56.1 ± 7.7	412.2 ± 129.1	1.2
		Flat Fan	−0.8 ± 0.1	46.6 ± 7.0	684.5 ± 262.3	2.0
	2,4-D	Unselected	−1.2 ± 0.1	71.9 ± 6.8	468.7 ± 83.5	—
		Air inclusion	−1.1 ± 0.1	78.4 ± 7.3	578.1 ± 114.4	1.2
		Flat Fan	−1.1 ± 0.1	85.5 ± 8.0	614.0 ± 116.1	1.3
	Dicamba	Unselected	−1.0 ± 0.1	29.9 ± 4.5	286.7 ± 63.0	—
		Air inclusion	−1.2 ± 0.2	37.4 ± 4.0	235.3 ± 46.5	0.8
		Flat Fan	−0.7 ± 0.1	33.8 ± 6.0	696.4 ± 181.5	2.4
Thayer	Glyphosate	Unselected	−1.4 ± 0.2	81.7 ± 12.5	402.8 ± 133.9	—
		Air inclusion	−0.8 ± 0.1	56.4 ± 9.1	984.6 ± 359.4	2.4
		Flat Fan	−1.0 ± 0.1	133.3 ± 22.5	1326.8 ± 374.3	3.3
	2,4-D	Unselected	−1.5 ± 0.2	78.3 ± 6.4	344.4 ± 56.2	—
		Air inclusion	−1.4 ± 0.2	156.0 ± 12.0	759.8 ± 131.4	2.2
		Flat Fan	−1.3 ± 0.1	101.3 ± 8.8	584.6 ± 106.2	1.7
	Dicamba	Unselected	−0.8 ± 0.2	19.7 ± 5.5	294.3 ± 93.2	—
		Air inclusion	−0.8 ± 0.1	27.8 ± 5.8	432.7 ± 121.5	1.5
		Flat Fan	−0.9 ± 0.1	62.6 ± 7.3	648.1 ± 147.5	2.2

^a^*b* parameter corresponds to the slope at the inflection point; *e* parameter corresponds to the inflection point; GR_90_ corresponds to the effective dose to reduce plant biomass by 90%; resistance ratios (R/S) were calculated as the ratio of the GR_90_ for each P_2_ population to the respective P_2_ unselected population.

The reduced herbicide sensitivity shifts reported herein are consistent with resistance shifts reported in previous recurrent selection studies with intentional applications of low herbicide rates. Glyphosate sensitivity shift of 2.15-fold in the lethal dose required to 95% control (LD_95_) was reported in a Palmer amaranth population recurrently selected for four generations with low rates of glyphosate^26^. Similar results were reported in an annual ryegrass population, where resistance ratios in the GR_50_ ranged from 1.68 to 1.87 in progenies recurrently selected with low rates of glyphosate^22^. A wild radish population had its 2,4-D sensitivity reduced 3.4-fold (LD_50_) after recurrent selection with low rates of 2,4-D following two selection rounds^18^. Moreover, authors reported a resistance shift of 8.6-fold as recurrent selection continued during two additional selection rounds. A similar trend was reported for a Palmer amaranth population recurrently selected with low rates of dicamba, where a 2.6-fold dicamba sensitivity shift (LD_90_) was reported following two rounds of selection^27^. Additionally, the authors reported a 3.9-fold dicamba resistance shift in the third selection round.

The 54.7-fold glyphosate sensitivity shift in the Palmer amaranth progeny from Perkins County is unprecedented in the literature. This large resistance shift indicates that although the initial Palmer amaranth progeny was classified as glyphosate-susceptible, individuals with a major glyphosate resistance mechanism were already present within the population prior to glyphosate drift selection. In a further screening, four out of 195 plants of the initial unselected Perkins population (P_0_) survived a diagnostic glyphosate rate (197 g ae ha^−1^). Investigations conducted at the Molecular Weed Science Laboratory at Colorado State University revealed that P_0_ plants from Perkins County that survived the 197 g ae ha^−1^ glyphosate rate had increased EPSPS copy number with 11 to 38 copies relative to ALS gene (low copy control gene) (supplementary information). The glyphosate resistance trait rapidly became predominant in this Palmer amaranth population following recurrent exposure to glyphosate drift.

Herbicide sensitivity reduction in this study varied across weed species, weed population, spray drift potential (nozzle), and herbicide active ingredient. In this study, waterhemp was more prone to herbicide sensitivity shifts following herbicide drift selection compared to Palmer amaranth. Moreover, the waterhemp population from Thayer County had more herbicide sensitivity shifts following herbicide drift selection compared to the Stanton County population. A similar trend was observed for Palmer amaranth, where the population from Perkins County was more prone to herbicide sensitivity reduction following herbicide drift selection compared to the population from Chase County. Across *Amaranthus* spp. populations tested herein, glyphosate sensitivity reduction was predominant over 2,4-D and dicamba following drift selection with the respective herbicides. Nozzle type influenced resistance shifts following herbicide drift exposure with glyphosate and dicamba, where progenies selected with the flat fan nozzle had greater selection intensity (higher mortality), and consequently larger resistance shifts. Interestingly, this trend was not observed for 2,4-D drift, where recurrent selection with the air inclusion nozzle resulted in slightly larger resistance shifts compared to the flat fan nozzle despite differences in selection intensity between nozzles.

Recurrent selection with low doses of herbicides can progressively favor for metabolism alleles present within the standing genetic variation of the population, which additively leads to non-target-site herbicide resistance^20,32^. A study reported that a previous susceptible annual ryegrass population evolved diclofop resistance following recurrent selection with low rates of diclofop^24^. Further investigations revealed that the recurrent selection with low rates of diclofop selected for non-target-site resistance with enhanced diclofop metabolism, likely mediated by cytochrome P450 monooxygenases (P450)^65^. A RNA-Seq transcriptome study with this population confirmed that not only P450 genes, but nitronate monooxygenase (NMO), glutathione transferase (GST), and glucosyltransferase (GT) genes were upregulated in diclofop-resistant plants^31^. Further studies reported upregulation of metabolic genes (GST) in a pyroxasulfone-resistant annual ryegrass population recurrently selected with low rates of the herbicide^19,30^. While Palmer amaranth and waterhemp populations with target-site glyphosate resistance (EPSPS gene amplification) were reported in Nebraska^66,67^, metabolic resistance to other herbicides is more frequent in waterhemp. A 2,4-D-resistant waterhemp population reported in Nebraska had rapid 2,4-D metabolism mediated by P450 enzymes^68^. Enhanced herbicide metabolism via P450 enzymes was also reported in a waterhemp population resistant to HPPD-inhibitor herbicides in Nebraska^69,70^. Atrazine resistance with rapid herbicide metabolism via enhanced GST conjugation was widespread in waterhemp populations in Nebraska^54^. Although non-target-site glyphosate resistance with metabolism in plants is relatively rare^71^, non-target-site resistance with reduced glyphosate translocation was identified in waterhemp biotypes in Mississippi^72^. Waterhemp biotypes with non-target-site resistance to glyphosate were also reported in Missouri^52^. Glyphosate metabolism with increased aldo-keto reductase (AKR) activity was reported in *Echinochloa colona* in Australia^73^.

Herbicide resistance alleles may be originally present within the standing genetic variation of the population or may immigrate via pollen or seeds from other populations^74^. As populations were collected in commercial cropping fields, and considering the rampant pollen-mediated gene flow and seeds transferring herbicide resistant alleles across waterhemp and Palmer amaranth populations in Nebraska, it can be inferred that herbicide resistance alleles could already be present within the standing genetic variation of the *Amaranthus* spp. populations tested herein^55,56^. This could explain the differences in herbicide sensitivity shift between waterhemp and Palmer amaranth, and even the differences among populations (different genetic background) following recurrent selection with herbicide drift. The influence of selection intensity (nozzle type), weed species, and weed population on glyphosate and dicamba sensitivity shifts following drift selection suggest that resistance alleles present within the standing genetic variability of populations were progressively selected during selection rounds. Some researchers suggest that low rates of herbicides could also act as stress agents inducing new stress-related mutations and epigenetic alterations that could ultimately lead to reduced herbicide sensitivity^33–35^. On the other hand, a study where over 70 million *Amaranthus hypochondriacus* L. seedlings were screened with imazethapyr showed no evidence suggesting that herbicide stress increased mutation rates conferring ALS resistance, although authors mentioned that stress-mediated increase of mutation rates leading to herbicide resistance remains biologically possible^74^. Both Perkins and Chase Palmer amaranth plants were physiologically stressed following dicamba drift and did not evolve levels of dicamba resistance following drift exposure for two generations, although we recognize that additional selection rounds would be necessary to expand the discussion. Interestingly, the 2,4-D sensitivity shifts in Palmer amaranth (Perkins) and waterhemp (Thayer) following drift selection were independent of selection intensity (nozzle type). Further studies are necessary to investigate the molecular basis of the sensitivity shifts found in the *Amaranthus* spp. following recurrent herbicide drift selection in this study.

The results of this study confirm that herbicide drift towards field margins can rapidly select for biotypes with reduced herbicide sensitivity with minor and major herbicide resistance mechanisms. Preventing the establishment of resistance prone weeds on field margins and ditches in agricultural landscapes is an important management strategy to delay herbicide resistance, especially for cross-pollinated weed species such as Palmer amaranth and waterhemp^16,75^. Weed management programs should consider strategies to mitigate near-field spray drift, and suppress weed populations on field borders and ditches in agricultural landscapes^16,62,75,76^.

## Supplementary Material

Supplementary Material.

## Data Availability

The datasets generated and/or analyzed during the current study are available from the corresponding author on reasonable request.
