# Supplementary material for: Herbicide drift exposure leads to reduced herbicide sensitivity in Amaranthus spp
Source: Sci Rep. 2020 Feb 7;10:2146. doi: 10.1038/s41598-020-59126-9 (PMC7005892; doi:10.1038/s41598-020-59126-9)
Supplement: Supplementary file 1 — Supplementary Material. [file 41598_2020_59126_MOESM1_ESM.pdf]

***EPSPS* gene copy number**

Genomic DNA was used to quantify 5-enolpyruvylshikimate-3-phosphate synthase (EPSPS) copy number with real-time quantitative PCR from Palmer amaranth plants (P0 unselected Perkins population) that survived the 197 g ae ha^{-1} glyphosate application [1,2]. Leaf tissue was collected from plants with DNEasy Plant Mini Kit (Qiagen, Valencia, CA) and quantified using a NanoDrop spectrophotometer (Thermo Scientific, Wilmington, DE). DNA concentrations were adjusted to 5 ng μl^{-1}, and primer sets and qPCR conditions were used as previously described (Gaines et al. 2010, Küpper et al. 2017). Threshold cycles for EPSPS and ALS were recorded by a CFX ConnectTM Real-Time PCR Detection System thermal cycler (Bio-Rad Laboratories, Hercules, CA). Relative EPSPS gene copy number was calculated as $2^{-\Delta Ct}$, with $\Delta Ct = [(Ct, ALS) - (Ct, EPSPS)]$[1,2]. Previously confirmed glyphosate-resistant and sensitive plants were included in the study for relative comparisons.

Results

Table 1. *EPSPS* copy number of Palmer amaranth plants (P0 unselected Perkins population) that survived 197 g ae of glyphosate ha^{-1}.

Sample	*EPSPS* copy number
Plant 1	38.2
Plant 2	21.3
Plant 3	16.1
Plant 4	11.1
Glyphosate-resistant control*	103.1
Glyphosate-susceptible control*	1.1

*Known glyphosate-susceptible and resistant plants used for relative comparisons.

References

1. Küpper, A. et al. Multiple Resistance to Glyphosate and Acetolactate Synthase Inhibitors in Palmer Amaranth (Amaranthus palmeri) Identified in Brazil. Weed Science 65, 317–326 (2017).

2. Gaines, T. A. et al. Gene amplification confers glyphosate resistance in Amaranthus palmeri. PNAS 107, 1029–1034 (2010).
